# Statistical Approach for Production of PUFA from *Kocuria* sp. BRI 35 Isolated from Marine Water Sample

**DOI:** 10.1155/2014/570925

**Published:** 2014-05-25

**Authors:** Swanandi Pote, Rama Bhadekar

**Affiliations:** Department of Microbial Biotechnology, Rajiv Gandhi Institute of I.T. and Biotechnology, Bharati Vidyapeeth Deemed University, Katraj, Pune, Maharashtra 411046, India

## Abstract

In this study, Plackett-Burman design was used to identify the most influential parameters affecting PUFA production by *Kocuria* sp. BRI 35 isolated from Antarctic water sample. Amongst 10 variables evaluated, magnesium chloride, protease peptone, glucose, and temperature were significant. Response surface methodology consisting of a central composite design was developed to study the interactions between the variables and to determine optimal values of significant variables. A quadratic model (*R* = 0.9652, *F* = 14.64, *P* < 0.0001) was built. The contour plots indicated that the isolate produced maximum PUFA at lower concentrations of magnesium sulfate (0.9 g/L) and higher concentrations of protease peptone (5 g/L) and glucose (10 g/L) at 15°C. MgSO_4_ and glucose exhibited quadratic as well as interactive effect on PUFA production whereas protease peptone and temperature showed interactive effects only. After optimization, PUFA production per unit biomass increased from 0.94 mg/g to 11.12 mg/g. This represented an increase from 3% to 58.62% of the total fatty acids. Among PUFAs, the yield of **ω**-6 fatty acids increased from 9.66 mg/L to 107.71 mg/L with significant increase in linoleic acid (20.36 mg/L) whereas **ω**-3 fatty acids increased up to 12.37 mg/L with DHA being the major **ω**-3 fatty acid produced.

## 1. Introduction

Research on production and purification of polyunsaturated fatty acids (PUFAs) has gained a lot of significance due to their role in human health and nutrition. PUFAs are fatty acids that have more than one double bond in their back bone structure. Based on the position of their double bonds they are grouped as *ω*-3, *ω*-6, and *ω*-9 fatty acids. Amongst these *ω*-3 fatty acids (alpha linolenic acid (ALA), eicosapentaenoic acid (EPA), and docosahexaenoic acid (DHA)) and *ω*-6 fatty acids (linoleic acid (LA), gamma linolenic acid (GLA), and arachidonic acid (AA)) are of importance in human health [1]. Moreover humans lack the enzymes required for synthesis of these acids [2]. Hence the only way to obtain these fatty acids is through nutrition. Deficiencies of *ω*-3 and *ω*-6 fatty acids are known to adversely affect the development and proper functioning of brain and central nervous system. Lack of these acids may lead to neurological disorders like attention deficit hyperactivity disorder (ADHD), dyslexia, dyspraxia, and autism [3]. Apart from neurological disorders PUFA deficiency has also been linked to various cardiovascular, cerebrovascular, autoimmune diseases and cancers [4]. *ω*-3 fatty acids are also known for their anti-inflammatory properties [5]. Widespread awareness about the benefit of PUFAs has led to increase in commercial production of PUFAs.

Fish oil is the most widely used source for obtaining PUFAs. However, this oil gets easily oxidized and hence is often associated with unpleasant taste and odor [6]. Besides, decrease in fish population and contamination of marine ecosystems with chemicals, heavy metals, and so forth are other factors that hamper the use of fish oil for PUFA production. Even though vegetable oils like corn oil, soybean oil, palm oil, and so forth provide an alternative source for PUFAs, they are often a complex mixture of various fatty acids [7]. Hence, extensive purification procedures become a necessity if these oils are to be used for obtaining PUFAs [8]. As a result, cost of PUFA production is greatly increased. In order to alleviate these problems other sources of PUFAs are now being explored. In light of this, microorganisms prove to be very promising alternative source. The superiority of microbes lies in the fact that they produce single PUFA in high quantities with high oxidative stability leading to reduced production and purification cost. Moreover, microorganisms are renewable source for PUFAs [9]. Thus, microbes not only surpass the disadvantages of other sources but also offer a quick and economical alternative for obtaining PUFAs.

Several fungi and bacteria are known to produce commercially important PUFAs. EPA production has been reported from* Moritella* (25 mg/g) [10] and* Pythium* (90 mg/L) [11], whereas* Thraustochytrium* [12] produces DHA (23 mg/L). Among *ω*-6 fatty acids, Klempova et al. [13] have reported GLA production (217 mg/L) from* Umbelopsis isabellina* CCF 2412. On the other hand* Mortierella alpina *produces AA (40–43% of total fatty acids) [14]. Bacteria especially from cold regions like Antarctica produce high amounts of PUFAs as an adaptation to the habitat [1]. However the arena of bacterial PUFAs is relatively new. To name a few,* Shewanella* species (10.3% of total fatty acids) [15],* Aureispira* sp. (21.5 mg/L of AA) [16], and* Photobacterium* sp. [17] are the examples of PUFA producing bacteria. Nichols et al. [18] have reviewed PUFA producing bacteria from the Antarctic region.

Considering importance of PUFA in human health and suitability of microbial sources, many researchers have published work on optimization of PUFA production from microorganisms [10, 19, 20]. Traditionally, a method of varying one parameter at a time has been employed for this. However, the method is time consuming and costly. The method does not evaluate the effect of interaction between variables and the influence of more than one variable simultaneously. Therefore, other statistical methods that allow such studies are preferred. The most commonly used statistical method for optimization of various parameters under study is response surface methodology (RSM). Use of RSM has great advantage since (i) significant variables can be easily identified, (ii) large amount of information about the system under study is obtained, (iii) it requires relatively few number of experiments, (iv) it allows interactive study of variables, (v) it is computationally less demanding, (vi) it predicts repose for conditions other than those under study, (vii) and it is less time consuming and economical [21]. So far several reports that highlight the use of RSM for optimization of PUFA production are available. Studies reporting use of RSM have been published by Zhou et al. [19] for DHA production from* Schizochytrium* sp., Elrazak et al. [20] for EPA production from marine bacteria, and so forth.

In this paper we report optimization of PUFA production from* Kocuria* sp. BRI 35. The most significant variables were identified using Plackett-Burman design. These variables were further optimized using RSM. To our knowledge this is the first report on optimization studies for PUFA production from* Kocuria *species.

## 2. Materials and Methods

### 2.1. Chemicals

All the media components and chemicals were purchased from Sigma Aldrich, Hi Media, and Merck (Mumbai, India) and were of analytical grade.

### 2.2. Organism

BRI 35 was isolated from marine water sample (latitude S 51°06′34.4′′ and longitude E 57°35′40.2′′). The isolate was maintained on marine salt medium (MSM) (composition per litre: 81.0 g NaCl, 10.0 g yeast extract, 9.6 g MgSO_4_, 7.0 g MgCl_2_, 5.0 g protease peptone, 2.0 g KCl, 1.0 g glucose, 0.36 g CaCl_2_, 0.06 g NaHCO_3_, 0.026 g NaBr, and 15 g agar with pH adjusted to 7.0  ±  0.2). BRI 35 was identified using 16S rRNA gene sequencing.

The organism was grown in MSM at 25°C and 120 rpm for 48 hours (h) and was further used for inoculation in all the experiments at 10% concentration. For measurement of microbial biomass (g/L) the cell pellets were lyophilized. For determination of lipid content, lipids in the cells were extracted, dried, and weighed according to method described by Bligh and Dyer [22]. Estimation of lipids was carried out using phosphovanillin reagent [23].

### 2.3. Analysis of PUFA Production

#### 2.3.1. Preparation of FAMEs

For all the experiments cell mass was obtained by centrifugation at 10,000 rpm for 10 minutes. 1 g of cell mass was boiled for 20 min in 0.5 N methanolic sodium hydroxide solution. Further 5 mL of boron trifluoride (methanolic) was added to the mixture. This was followed by vortexing and 15 minutes of incubation. The FAMEs thus prepared were extracted in 1 mL heptane [24].

#### 2.3.2. GC-FID for FAMEs

A gas chromatograph equipped with a 60 m × 250 *μ*m × 0.25 *μ*m gas capillary column and a flame ionization detector was employed for FAMEs analyses. Nitrogen was used as a carrier gas at a pressure of 19.865 psi. 1 *μ*L of sample was injected using split mode. Initial temperature of the column was 110°C which was increased up to 250°C at a rate of 3°C per minute. The column was maintained at 250°C for 2 minutes. The fatty acids present were detected by comparison of the retention time with those of standards.

#### 2.3.3. Determination of Significant Variables for PUFA Production

The significant variables affecting PUFA production from* Kocuria* sp. BRI 35 were identified using the Plackett-Burman design. In all, 8 nutritional factors, namely, sodium chloride (NaCl), yeast extract (YE), magnesium sulfate (MgSO_4_), magnesium chloride (MgCl_2_), protease peptone (P.Pep), potassium chloride (KCl), glucose, and calcium chloride (CaCl_2_), along with 2 physical parameters, namely, temperature and pH, were evaluated. Each factor was studied at two levels. The variables studied and their high (+1) and low levels (−1) are presented in Table 1. A design for 12 experiments was generated using Design-Expert version 8.0 (Stat-Ease, Inc., Minneapolis, USA) software (Table 2). An additional experiment where the variables were maintained at values equal to those in MSM was included in the design along with the standard 12 experiments. The organism was cultivated in 100 mL medium in 250 mL Erlenmeyer flasks for 48 hours. All the experiments were carried out in triplicate and the response was measured in terms of the amount of PUFA produced by the organism. The variables with* P* value < 0.05 were considered to be significant.

#### 2.3.4. Central Composite Design (CCD)

The significant variables identified using the Plackett-Burman design were optimized using response surface methodology. A central composite design (CCD) matrix for 4 significant variables was generated. The total number of experiments generated was 2^*k*^ + 2*K* + *n*_0_ (where *k* is the number of independent variables and *n*_0_ is the number of experiments carried out at central point values of variables). The matrix consisted of 6 central points and included experiments where one variable was set at extreme ±2 level and the other variables were maintained at central point level. In experimental runs where the software generated negative values, that component was not added in the medium.  Table 4 summarizes the coded levels of the variables and their actual values.  Table 5 gives the design matrix and the response, that is, amount of PUFAs produced. The coding of the variables was done in accordance with the following equation:

(1) $$\begin{array}{c} y_{c}=\frac{Yc-Ycp}{\Delta yc}, \end{array}$$

where *y*_*c*_ is coded level, *Yc* is actual value, *Yc* *p* is real value of central point, and Δ*yc* is the step change. The amount of PUFAs produced can be expressed by the quadratic equation

(2) $$\begin{array}{c} X=\beta_{0}+\sum \beta_{c}y_{c}+\sum \beta_{cc}y_{c}^{2}+\sum \beta_{cb}y_{c}y_{b}, \end{array}$$

where *X* is predicted response; *β*_0_ is the intercept; *β*_*c*_, *β*_*cc*_, and *β*_*cb*_ are linear, quadratic, and interactive coefficients, respectively.

The responses generated were analyzed using Design-Expert version 8.0. They were subjected to multiple regression analysis for calculation of the coefficients. The significance of the model was determined for testing its efficiency. The model under study was considered to be fit for optimization if it had a significant *F* value and a good multiple correlation coefficient (*R*). The fermentation conditions to obtain maximum PUFA production from BRI 35 were predicted using numerical optimization in the software. The factors under study were varied and the remaining variables were kept at level equal to those in MSM.

## 3. Results and Discussion

### 3.1. Organism

BRI 35 was maintained on MSM slants. The DNA was extracted using standard protocols and the isolate was identified using 16S rRNA gene sequencing. The results indicated it belonged to the genus* Kocuria* (99% similarity, 1288 bp) (GenBank accession number: KF366396) [25].

### 3.2. Determination of Significant Variables for PUFA Production

Plackett-Burman design was used to identify the significant variables affecting PUFA production from* Kocuria* sp. BRI 35. Eight nutritional and two physical parameters were studied at high and low levels. Table 2 demonstrates the experimental design and the response generated in terms of the amount of PUFAs produced. The effect of each variable was determined by the following equation:

(3) $$\begin{array}{c} X=\frac{2[\sum R^{+}-R^{-}]}{N}, \end{array}$$

where *X* is the effect of the tested variable, *R* ^+^ and *R* ^−^ are the responses for high and low values, respectively, and *N* is the total number of experiments. The final equation generated in terms of actual factors was

(4) R=+4.90846+1.13250∗NaCl−1.21750∗YE +2.29917∗MgSO4+1.97083∗MgCl2 −2.35417∗P.Pep+0.26083∗KCl +1.59917∗CaCl2+2.89750∗Glucose −3.24083∗Temperature+0.027500∗pH.

The results thus obtained were subjected to statistical analysis using Design-Expert software. The variables were segregated on the basis of their *P* value at the confidence level of 95% (*P* value <0.05) and those with *P* value <0.05 were considered to be significant.  Table 3 shows analysis of variance (ANNOVA) for PUFA production. Based on the results of ANNOVA, MgSO_4_ (*P* = 0.0406), protease peptone (*P* = 0.0388), glucose (*P* = 0.0261), and temperature (*P* = 0.0211) were identified as variables that significantly affect PUFA production from* Kocuria* sp. BRI 35. On the other hand, variables like NaCl, YE, MgCl_2_, CaCl_2_, and pH exhibited *P* values >0.05 and hence were considered to be insignificant for PUFA production. Literature review suggests that amount of PUFA produced by microorganisms is greatly influenced by the temperature at which the microorganism is cultivated. Generally, microorganisms tend to produce more amounts of PUFAs at lower temperatures (5–25°C) [2, 26, 27]. Along with temperature, the amount of carbon and nitrogen sources, that is, C : N ratio [28], and the concentrations of salts like MgSO_4_, CaCl_2_, and so forth in the medium are also known to affect the type and amount of PUFAs produced [29]. Our observations are in-line with these findings. The significant parameters thus identified were selected for further optimization of PUFA production.

### 3.3. Central Composite Design

Response surface methodology was used to optimize the concentrations of the significant parameters identified by Plackett-Burman design. In order to evaluate the interactive effect of the variables, experiments involving different combinations of variables were designed. Accordingly, CCD matrix consisting of a factorial design with 6 replications of the central point was developed. Multiple regression analysis of the experimental data generated the following equation for PUFA production in terms of actual factors:

(5) R1=−23.76481−5.53477∗MgSO4+27.97817∗P.Pep −1.82401∗Glucose+2.86810∗Temperature −0.86424∗MgSO4∗P.Pep−0.27950∗MgSO4 ∗Glucose+0.38270∗MgSO4∗Temperature −0.34457∗P.Pep∗Glucose−1.10367∗P.Pep ∗Temperature+0.031056∗Glucose∗Temperature +0.14875∗MgSO42+0.13700∗P.Pep2+0.32258 ∗Glucose2−0065558∗Temperature2.

The statistical significance of the quadratic model built was studied by *F*-test and analysis of variance (ANNOVA) (Table 6). The efficiency of the model was determined by studying its characteristics. The model was found to have *F* value of 14.64 indicating that there is only 0.01% chance that such a high model *F* value could occur due to noise. The *P* value of the model was less than 0.0001. This clearly identifies a reliability of 99.9%. Multiple regression coefficient *R* and coefficient *R* ^2^ of the model were also determined. The closer the value of *R* to 1, the better the correlation between experimental and predicted values [30, 31]. In the case of this model, multiple regression coefficient *R* had a value of 0.9652 illustrating a very good correlation between the experimental and predicted values (Table 5). The *R* ^2^ was 0.9318 whereas the value of adjusted *R* ^2^ was 0.9078. This suggests that around 94% of variation is due to the independent variables and only 6% of the variation cannot be explained by the model. Also the standard deviation was 5.91 which is less than 10. Hence, the model is highly significant and can be efficiently used for response prediction.

**Figure 1:**
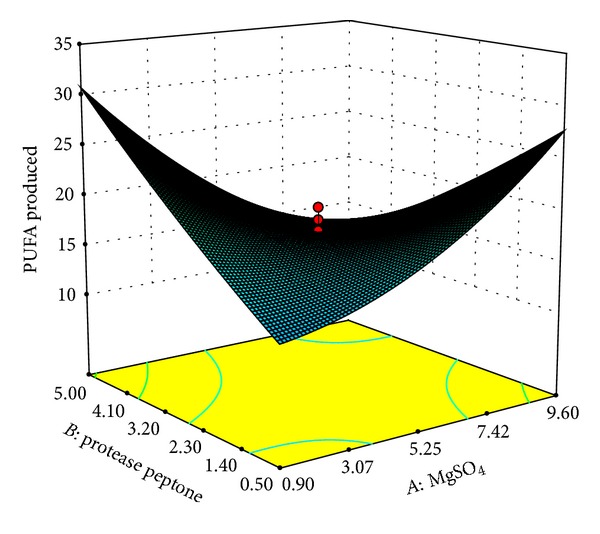
Response surface plot of PUFAs produced (% of total fatty acids) as a function of MgSO_4 _(g/L) and protease peptone (g/L).

**Figure 2:**
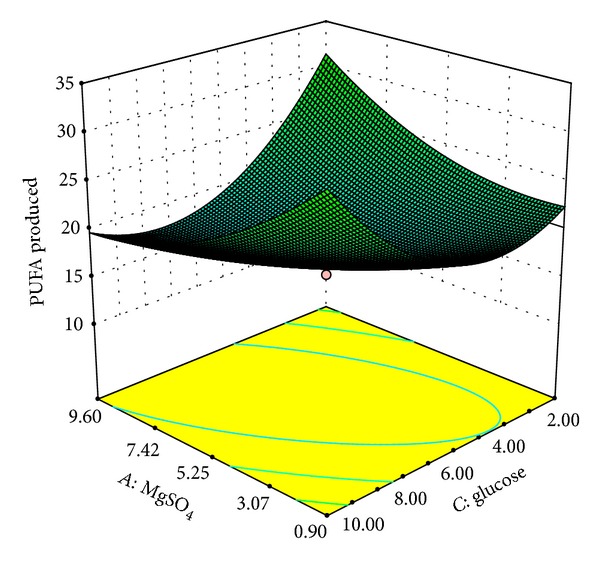
Response surface plot of PUFAs produced (% of total fatty acids) as a function of MgSO_4_(g/L) and glucose (g/L).

**Figure 3:**
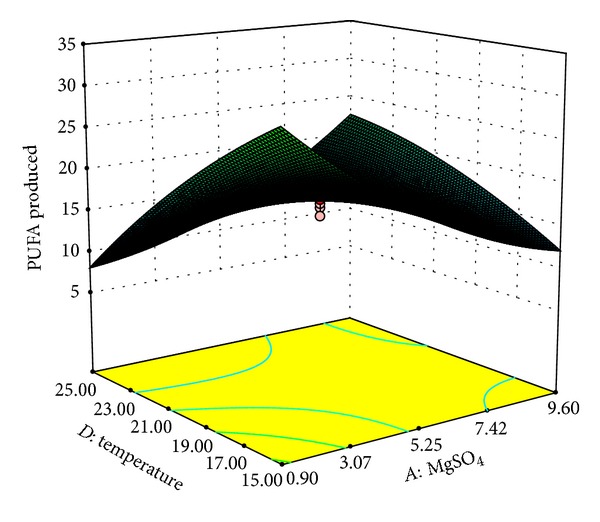
Response surface plot of PUFAs produced (% of total fatty acids) as a function of MgSO_4_(g/L) and temperature (°C).

Our results indicated that variables *A* and *C* had not only quadratic (*A* ^2^: *P* < 0.0247; *C* ^2^: *P* < 0.001) but also interactive effect on PUFA production (Table 6). On the other hand variables *B* and *D* were found to have interactive effects only. Amongst the interactive effects, interaction between *AB*, *AD*, and *BD* (*P* < 0.0001) was found to be the most significant followed by *AC* (*P* < 0.0021) and *BC* (*P* < 0.0321). The optimal values of the variables were determined by constructing three-dimensional plots. The shapes of the plots are suggestive of the significance of the interaction between variables with respect to the response obtained. Elliptical plots indicate a significant interaction whereas circular plots indicate that the interaction between the variables is not a considerable contributor to the response obtained [32]. Here, % PUFAs obtained were plotted as function of significant interactive variables. The interactive effects of MgSO_4_ (*A*) with protease peptone (*B*), glucose (*C*), and temperature (*D*) on PUFA production are demonstrated in Figures 1, 2, and 3, respectively. The plots clearly indicate that lower concentrations of MgSO_4_ are beneficial for PUFA production from* Kocuria* sp. BRI 35 with maximum production at 0.9 g/L MgSO_4_. Kang et al. [33] have reported maximum overall PUFA production (67.10%) in medium without MgSO_4_ from* Thraustochytrium aureum* ATCC 34304. However, the same bacterium produces DHA optimally at 4.5 g/L of MgSO_4_ [34]. Thus, MgSO_4_ plays a critical role in overall PUFA production as well as synthesis of individual fatty acids. This may be attributed to its function as a cofactor for enzymes involved in fatty acid synthesis. Hence its addition at proper concentration may increase the efficiency of the enzymes involved in fatty acid synthesis [35].

**Figure 4:**
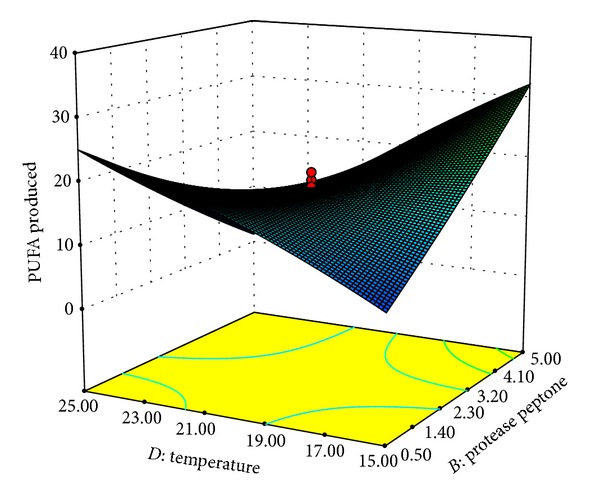
Response surface plot of PUFAs produced (% of total fatty acids) as a function of protease peptone (g/L) and temperature (°C).

The interactive effects of temperature (*D*) with MgSO_4_ (*A*) and protease peptone (*B*) are shown in Figures 3 and 4, respectively. As depicted in the plots maximum PUFA production is observed at lower temperature of around 15°C. The amount of PUFA produced was found to decrease considerably with increase in temperature. Similarly, Nichols et al. [36] have reported maximum EPA (12.2%) production at 15°C from a psychrophilic bacterium isolated from the Antarctic sea. Optimum temperature of 10°C was observed by Chodok et al. [2] for overall PUFA production from* Physcomitrella patens*.

**Figure 5:**
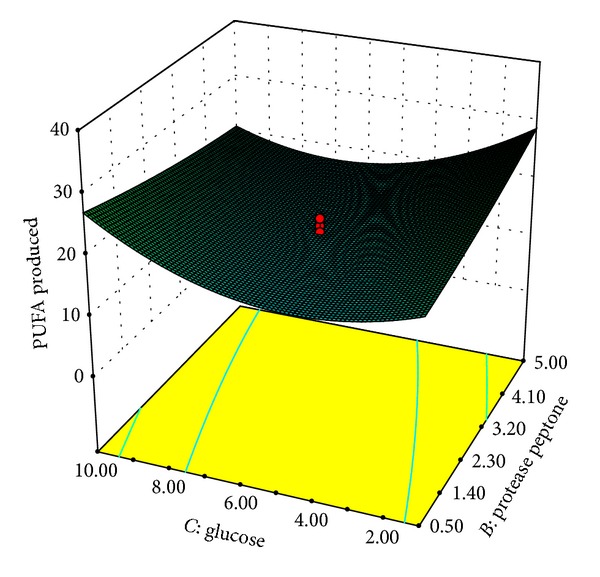
Response surface plot of PUFAs produced (% of total fatty acids) as a function of protease peptone (g/L) and glucose (g/L).

Figure 5 shows the interactive effect of protease peptone (*B*) and glucose (*C*) on PUFA synthesis from BRI 35. The isolate produced PUFAs optimally in presence of protease peptone and glucose at 5 g/L and 10 g/L concentration, respectively. Similarly, Jang et al. [28] have reported optimum C : N ratio of 2 : 1 for PUFA production from* Mortierella alpina*. However authors have used starch as carbon source and KNO_3_ and yeast extract as nitrogen sources. C : N ratio of 25 : 7 was found to be optimum for production of ARA from* Mortierella alpina *CBS 754.68 [37]. Higher C : N ratio (5 : 1) for DHA production had been reported by Wu et al. from* Schizochytrium *sp. S31 [38].

Thus, analysis of RSM plots indicated that maximum PUFAs are obtained from* Kocuria* sp. BRI 35 under the following fermentation conditions: MgSO_4_ 0.94 g/L; protease peptone 5 g/L; glucose 10 g/L; temperature 15°C. The optimization strategies resulted in significant increase in the amount of PUFAs produced. In original medium PUFAs represented only 3% of total fatty acids produced. This corresponded to 0.94 mg/g of biomass. In the optimized medium more than 50% of fatty acids produced were PUFAs which accounted for 11.12 mg/g of biomass. Thus, the amount of PUFA's increased from 9.66 mg/L to 120.08 mg/L (Table 7).* Kocuria* sp. BRI 35 exhibited the ability to produce higher amounts of PUFAs in optimized medium without significant increase in the overall lipid content. Earlier Chodok et al. have reported maximum PUFA production of 75.11 mg/L by* Physcomitrella patens* in medium optimized using Plackett-Burman design [2]. Use of sugarcane molasses for PUFA production has been documented by Li et al. They have reported PUFA production of 5.74 g/L from* Mucor recurvus* under optimized conditions [39]. Presence of increased amounts of PUFAs from* Kocuria* sp. BRI 35 (this work) in optimized medium is a unique finding since saturated and monounsaturated fatty acids have been mainly reported from the genus* Kocuria *[40, 41].

Table 8 demonstrates % increase and quantity (mg/L) of individual PUFAs produced from BRI 35 following optimization. The amount of *ω*-6 fatty acids increased from 9.66 mg/L (3% of total fatty acids) to 120.08 mg/L (50.23% of total fatty acids). Specifically, yield of linoleic acid increased from 1.14 mg/L to 20.36 mg/L under optimized conditions. Along with *ω*-6 fatty acids, our isolate exhibited the ability to produce *ω*-3 fatty acids particularly DHA (11.28 mg/L) under optimal conditions. Similarly, Morita et al. have reported maximum DHA production of 13.4 mg/L from* Moritella* sp. [42]. Along with DHA and LA* Kocuria* sp. BRI 35 also produced several beneficial *ω*-6 (ARA, GLA) and *ω*-3 (EPA, ALA) PUFAs (Table 8).

## 4. Conclusion

Optimization of culture conditions for production of PUFAs from* Kocuria* sp. BRI 35 was accomplished using Plackett-Burman design and response surface methodology. MgSO_4_, protease peptone, glucose, and temperature were found to be the most significant parameters. Under optimized conditions, BRI 35 produced nutritionally important PUFAs like EPA, DHA, ARA, LA, GLA, and ALA. Thus BRI 35 exhibits the potential for commercial production of PUFAs.

## Figures and Tables

**Table 1:** Variables studied using Plackett-Burman design.

Variable	Code	High value (+1)	Low value (−1)
NaCl (g/L)	Y1	80	40
Yeast extract (YE) (g/L)	Y2	10	1
MgSO_4_ (g/L)	Y3	9.6	0.9
MgCl_2_ (g/L)	Y4	7	0.7
Protease peptone (P.Pep.) (g/L)	Y5	5	0.5
KCl (g/L)	Y6	2	0.2
Glucose (g/L)	Y7	10	1
CaCl_2_ (g/L)	Y8	0.3	0.03
Temperature (°C)	Y9	25	15
pH	Y10	8.5	6.5

**Table 2:** Plackett-Burman design for evaluating the significant variables for PUFA production by *Kocuria* sp. BRI 35.

Run	Y1	Y2	Y3	Y4	Y5	Y6	Y7	Y8	Y9	Y10	Dummy	PUFA production (% of total fatty acids) [mean ± standard error]
1	−1	−1	−1	−1	−1	−1	−1	−1	−1	−1	−1	20.5 ± 0.56
2	+1	+1	−1	−1	−1	+1	−1	+1	+1	−1	+1	1.0 ± 0.16
3	+1	+1	−1	+1	+1	+1	−1	−1	−1	+1	−1	1.69 ± 0.08
4	+1	−1	+1	+1	+1	−1	−1	−1	+1	−1	+1	0.95 ± 0.20
5	−1	+1	+1	+1	−1	−1	−1	+1	−1	+1	+1	13.29 ± 0.62
6	0	0	0	0	0	0	0	0	0	0	0	3.0 ± 0.06
7	−1	+1	−1	+1	+1	−1	+1	+1	+1	−1	−1	1.36 ± 0.41
8	+1	−1	+1	+1	−1	+1	+1	+1	−1	−1	−1	22.37 ± 0.61
9	−1	+1	+1	−1	+1	+1	+1	−1	−1	−1	+1	2.51 ± 0.59
10	−1	−1	−1	+1	−1	+1	+1	−1	+1	+1	+1	2.57 ± 0.26
11	+1	−1	−1	−1	+1	−1	+1	+1	−1	+1	+1	7.94 ± 0.68
12	−1	−1	+1	−1	+1	+1	−1	+1	+1	+1	−1	1.83 ± 0.50
13	+1	+1	+1	−1	−1	−1	+1	−1	+1	+1	−1	3.25 ± 0.47

**Table 3:** Statistical analysis of Plackett-Burman design.

Variables	Coefficient	*P* value
NaCl	1.13	0.1413
Yeast extract	−1.22	0.1256
MgSO_4_	2.30	**0.0406**
MgCl_2 _	1.97	0.0541
Protease peptone	−2.35	**0.0388**
KCl	0.26	0.6399
CaCl_2_	1.60	0.0789
Glucose	2.90	**0.0261**
Temperature	−3.24	**0.0211**
pH	0.028	0.9593

**Table 4:** Coded and real values of variables selected for CCD.

Variable	Symbol	Unit	Coded levels				
			−2	−1	0	+1	+2
MgSO_4_	*A*	g/L	−3.45	0.90	5.25	9.60	13.95
Protease Peptone	*B*	g/L	−1.75	0.50	2.75	5.0	7.25
Glucose	*C*	g/L	−3.50	1.00	5.50	10	14.50
Temperature	*D*	°C	10	15	20	25	30

**Table 5:** CCD matrix of variables with response.

Run	MgSO_4_	Protease peptone	Glucose	Temperature	PUFA production	
					(% of total fatty acids)	
					[Mean ± standard error]	
					Actual values	Predicted values
1	−1	+1	−1	−1	57.86 ± 0.92	58.45
2	−1	−1	−1	+1	10.11 ± 0.96	9.48
3	+1	+1	−1	−1	38.13 ± 1.19	33.80
4	0	0	0	+2	11.78 ± 0.79	4.6
5	0	0	0	0	17.26 ± 0.34	17.25
6	+1	+1	+1	−1	6.53 ± 0.46	13.86
7	+1	−1	+1	−1	18.57 ± 0.60	11.90
8	0	0	0	0	16.78 ± 0.85	17.25
9	+1	+1	+1	+1	2.32 ± 0.30	0.98
10	0	0	0	0	19.6 ± 0.52	17.25
11	0	0	+2	0	44.81 ± 0.86	42.76
12	−1	−1	−1	−1	9.97 ± 0.09	8.70
13	+1	+1	−1	+1	7.96 ± 0.05	18.13
14	−1	+1	+1	+1	11.42 ± 0.29	14.22
15	+2	0	0	0	29.81 ± 0.41	26.49
16	+1	−1	+1	+1	42.66 ± 0.86	48.69
17	−2	0	0	0	31.25 ± 0.19	30.54
18	0	−2	0	0	14.52 ± 0.20	19.01
19	+1	−1	−1	+1	56.26 ± 0.45	51.88
20	0	+2	0	0	29.57 ± 0.55	21.04
21	−1	−1	+1	+1	26.37 ± 0.68	28.09
22	0	0	0	0	16.29 ± 0.24	17.25
23	−1	+1	+1	−1	58.62 ± 0.35	60.39
24	0	0	0	0	18.35 ± 0.51	17.25
25	0	0	−2	0	45.99 ± 0.49	44.01
26	0	0	0	0	15.28 ± 0.42	17.25
27	+1	−1	−1	−1	14.07 ± 0.08	17.89
28	−1	+1	−1	+1	5.42 ± 0.52	9.48
29	−1	−1	+1	−1	28.15 ± 1.70	24.60
30	0	0	0	−2	13.65 ± 0.28	16.79

**Table 6:** ANNOVA for quadratic model.

Source	Sum of squares	df	*F* value	*P* value
Model	7151.88	14	14.64	**<0.0001**
*A*	24.60	1	0.71	0.4142
*B*	6.20	1	0.18	0.6793
*C*	2.34	1	0.067	0.7990
*D*	222.77	1	6.39	**0.0232**
*AB*	1144.81	1	32.81	**<0.0001**
*AC*	478.95	1	13.73	**0.0021**
*AD*	1108.56	1	31.77	**<0.0001**
*BC*	194.74	1	5.58	**0.0321**
*BD*	2466.61	1	70.70	**<0.0001**
*CD*	7.81	1	0.22	0.6429
*A* ^2^	217.32	1	6.23	**0.0247**
*B* ^2^	13.19	1	0.38	0.5478
*C* ^2^	1170.40	1	33.55	**<0.0001**
*D* ^2^	73.68	1	2.11	0.1668

**Table 7:** Increase in PUFA production after optimization.

Design	% PUFA produced [mean ± standard error]	PUFA produced (mg/L)	Dry cell weight (g/L)	PUFA produced per unit biomass (mg/g)
Original medium	3.0 ± 0.06	9.66	10.3	0.94
RSM	58.62 ± 0.35	120.08	10.8	11.12

**Table 8:** Yield of *ω*-3/*ω*-6 fatty acids produced in MSM and optimized medium.

Fatty acids											
	*ω*-6						*ω*-3				
	18:2 (trans)	18:2 (cis)	18:3	20:2	20:3	20:4	22:2	18:3	20:3	20:5	22:6
	MSM										
PUFAs (% of total fatty acids)	0.85 ± 0.42	0.56 ± 0.35	0.0	0.0	1.06 ± 0.55	0.53 ± 0.09	0.0	0.0	0.0	0.0	0.0
PUFAs produced (mg/L)	0.67	1.14	0.0	0.0	4.30	3.55	0.0	0.0	0.0	0.0	0.0

	Optimized medium										
PUFAs (% of total fatty acids)	44.26 ± 0.56	5.01 ± 0.38	0.49 ± 0.04	0.0	0.0	0.23 ± 0.15	0.0	0.24 ± 0.08	0.14 ± 0.05	0.06 ± 0.02	8.19 ± 0.50
PUFAs produced (mg/L)	84	20.36	2.60	0.0	0.0	0.75	0.0	0.77	0.22	0.1	11.28

## References

[1] Jadhav VV, Yadav A, Shouche YS, Bhadekar RK (2013). Isolation and cellular fatty acid composition of psychrotrophic *Halomonas* strains from Antarctic sea water. *Songklanakarin Journal of Science and Technology*.

[2] Chodok P, Kanjana-Opas A, Kaewsuwan S (2010). The plackett-burman design for evaluating the production of polyunsaturated fatty acids by *Physcomitrella patens*. *Journal of the American Oil Chemists’ Society*.

[3] Schuchardt JP, Huss M, Stauss-Grabo M, Hahn A (2010). Significance of long-chain polyunsaturated fatty acids (PUFAs) for the development and behaviour of children. *European Journal of Pediatrics*.

[4] Su K-P (2008). Mind-body interface: the role of n-3 fatty acids in psychoneuroimmunology, somatic presentation, and medical illness comorbidity of depression. *Asia Pacific Journal of Clinical Nutrition*.

[5] Calder PC (2013). Omega-3 polyunsaturated fatty acids and inflammatory processes: nutrition or pharmacology?. *British Journal of Clinical Pharmacology*.

[6] Maqsood S, Benjakul S, Kamal-Eldin A (2012). Extraction, processing, and stabilization of health-promoting fish oils. *Recent Patents on Food, Nutrition, and Agriculture*.

[7] Turchini GM, Francis DS, Senadheera SPSD, Thanuthong T, de Silva SS (2011). Fish oil replacement with different vegetable oils in Murray cod: evidence of an “omega-3 sparing effect” by other dietary fatty acids. *Aquaculture*.

[8] Sijtsma L, de Swaaf ME (2004). Biotechnological production and applications of the *ω*-3 polyunsaturated fatty acid docosahexaenoic acid. *Applied Microbiology and Biotechnology*.

[9] Jadhav VV, Jamle MM, Pawar PD, Devare MN, Bhadekar RK (2010). Fatty acid profiles of PUFA producing Antarctic bacteria: correlation with RAPD analysis. *Annals of Microbiology*.

[10] Jacobs A, Botha A, van Zyl WH (2009). The production of eicosapentaenoic acid by representatives of the genus *Mortierella* grown on brewers’ spent grain. *Biologia*.

[11] Athalye SK, Garcia RA, Wen Z (2009). Use of biodiesel-derived crude glycerol for producing eicosapentaenoic acid (EPA) by the fungus *Pythium irregulare*. *Journal of Agricultural and Food Chemistry*.

[12] Shene C, Leyton A, Rubilar M, Pinelo M, Acevedo F, Morales E (2013). Production of lipids and docosahexasaenoic acid (DHA) by a native *Thraustochytrium* strain. *European Journal of Lipid Science and Technology*.

[13] Klempova T, Basil E, Kubatova A, Certik M (2013). Biosynthesis of gamma-linolenic acid and beta-carotene by *Zygomycetes* fungi. *Biotechnology Journal*.

[14] Dedyukhina EG, Chistyakova TI, Kamzolova SV, Vinter MV, Vainshtein MB (2012). Arachidonic acid synthesis by glycerol-grown*Mortierella* alpine. *European Journal of Lipid Science and Technology*.

[15] Hirota K, Nodasaka Y, Orikasa Y, Okuyama H, Yumoto I (2005). *Shewanella pneumatophori* sp. nov., an eicosapentaenoic acid-producing marine bacterium isolated from the intestines of Pacific mackerel (*Pneumatophorus japonicus*). *International Journal of Systematic and Evolutionary Microbiology*.

[16] Saelao S, Kanjana-Opas A, Kaewsuwan S (2011). Optimization of biomass and arachidonic acid production by *Aureispira maritima* using response surface methodology. *Journal of the American Oil Chemists’ Society*.

[17] Allen EE, Bartlett DH (2002). Structure and regulation of the omega-3 polyunsaturated fatty acid synthase genes from the deep-sea bacterium *Photobacterium profundum* strain SS9. *Microbiology*.

[18] Nichols DS, Nichols PD, McMeekin TA (1993). Polyunsaturated fatty acids in Antarctic bacteria. *Antarctic Science*.

[19] Zhou L, Lu Y, Zhou M, Zhao X (2007). Enhanced production of docosahexaenoic acid using *Schizochytrium* sp. by optimization of medium components. *Journal of Chemical Engineering of Japan*.

[20] Elrazak AA, Ward AC, Glassey J (2013). Response surface methodology for optimizing the culture conditions for eicosapentaenoic acid production by marine bacteria. *Journal of Industrial Microbiology & Biotechnology *.

[21] Wani TA, Ahmad A, Zargar S, Khalil NY, Darwish IA (2012). Use of response surface methodology for development of new microwell-based spectrophotometric method for determination of atrovastatin calcium in tablets. *Chemistry Central Journal*.

[22] Bligh E, Dyer W (1959). A rapid method of total lipid extraction and purification. *Canadian Journal of Biochemistry and Physiology*.

[23] Jadhav VV, Salunkhe DS, Bhadekar RK (2012). Effect of alterations in conventional medium on lipid accumulation and fatty acid content in oleaginous yeast. *International Journal of Pharmacy and Biological Sciences B*.

[24] Ichihara K, Fukubayashi Y (2010). Preparation of fatty acid methyl esters for gas-liquid chromatography. *Journal of Lipid Research*.

[25] Pote SS, Chaudhary Y, Upadhayay S

[26] Yang S-H, Lee J-H, Ryu J-S, Kato C, Kim S-J (2007). *Shewanella donghaensis* sp. nov., a psychrophilic, piezosensitive bacterium producing high levels of polyunsaturated fatty acid, isolated from deep-sea sediments. *International Journal of Systematic and Evolutionary Microbiology*.

[27] Allen EE, Facciotti D, Bartlett DH (1999). Monounsaturated but not polyunsaturated fatty acids are required for growth of the deep-sea bacterium *Photobacterium profundum* SS9 at high pressure and low temperature. *Applied and Environmental Microbiology*.

[28] Jang H-D, Lin Y-Y, Yang S-S (2005). Effect of culture media and conditions on polyunsaturated fatty acids production by *Mortierella alpina*. *Bioresource Technology*.

[29] Furlan VM, Paulo M, Batista I, Bandarra NM, Santo MLE, Prentice C (2012). Effect of the concentration of glucose in the docosahexaenoic acid (DHA) production by*Thraustochytrium* sp., ATCC, 26185. *Advance Journal of Food Science and Technology*.

[30] Wang Y-X, Lu Z-X (2005). Optimization of processing parameters for the mycelial growth and extracellular polysaccharide production by *Boletus* spp. ACCC 50328. *Process Biochemistry*.

[31] Vasconcelos AFD, Barbosa AM, Dekker RFH, Scarminio IS, Rezende MI (2000). Optimization of laccase production by *Botryosphaeria* sp. in the presence of veratryl alcohol by the response-surface method. *Process Biochemistry*.

[32] Muralidhar RV, Chirumamila RR, Marchant R, Nigam P (2001). A response surface approach for the comparison of lipase production by *Candida cylindracea* using two different carbon sources. *Biochemical Engineering Journal*.

[33] Kang D-H, Jeh E-J, Seo J-W, Chun B-H, Hur B-K (2007). Effect of salt concentration on production of polyunsaturated fatty acids in *Thraustochytrium aureum* ATCC 34304. *Korean Journal of Chemical Engineering*.

[34] Min KH, Lee HH, Anbu P, Chaulagain BP, Hur BK (2012). The effects of culture condition on the growth property and docosahexaenoic acid production from *Thraustochytrium aureum* ATCC, 34304. *Korean Journal of Chemical Engineering*.

[35] Muhid F, Nawi WNNW, Kader AJA, Yusoff WMW, Hamid AA (2008). Effects of metal ion concentrations on lipid and gamma linolenic acid production by *Cunninghamella* sp. 2A1. *OnLine Journal of Biological Sciences*.

[36] Nichols DS, Brown JL, Nichols PD, McMeekin TA (1997). Production of eicosapentaenoic and arachidonic acids by an Antarctic bacterium: response to growth temperature. *FEMS Microbiology Letters*.

[37] Rocky-Salimi K, Hamidi-Esfahani Z, Abbasi S (2011). Statistical optimization of arachidonic acid production by *Mortierella alpina* CBS 754.68 in submerged fermentation. *Iranian Journal of Biotechnology*.

[38] Wu S-T, Yu S-T, Lin L-P (2005). Effect of culture conditions on docosahexaenoic acid production by *Schizochytrium* sp. S31. *Process Biochemistry*.

[39] Li N, Deng Z-N, Qin Y-L, Chen C-L, Liang Z-Q (2008). Production of polyunsaturated fatty acids by *Mucor recurvus* sp. with sugarcane molasses as the carbon source. *Food Technology and Biotechnology*.

[40] Reddy GSN, Prakash JSS, Prabahar V, Matsumoto GI, Stackebrandt E, Shivaji S (2003). *Kocuria polaris* sp. nov., an orange-pigmented psychrophilic bacterium isolated from an Antarctic cyanobacterial mat sample. *International Journal of Systematic and Evolutionary Microbiology*.

[41] Kim SB, Nedashkovskaya OI, Mikhailov VV (2004). *Kocuria marina* sp. nov., a novel actinobacterium isolated from marine sediment. *International Journal of Systematic and Evolutionary Microbiology*.

[42] Morita N, Nishida T, Tanaka M, Yano Y, Okuyama H (2005). Enhancement of polyunsaturated fatty acid production by cerulenin treatment in polyunsaturated fatty acid-producing bacteria. *Biotechnology Letters*.

